# Traffic Light Coding System for Engaging With AI in Surgery

**DOI:** 10.1111/ans.70172

**Published:** 2025-05-15

**Authors:** Payal Mukherjee, Amin Beheshti, Shivani Angelique Kumar, Gordon Wallace, Neil Merrett, Jonathan Clark, Simon Kos, Ellen Rawstron, Jian Yang, Stuart Grieve, Amith Shetty, Simon Singer

**Affiliations:** ^1^ Faculty of Medicine, Health and Human Sciences Macquarie University Sydney Australia; ^2^ Royal Prince Alfred Institute of Academic Surgery, Royal Prince Alfred Hospital Sydney Australia; ^3^ Sydney Medical School, Faculty of Health and Medicine The University of Sydney Sydney Australia; ^4^ Department of Head and Neck Surgery Chris O'brien Lifehouse Sydney Australia; ^5^ Centre for Applied Artificial Intelligence, Macquarie University Sydney Australia; ^6^ Australian Institute for Innovative Materials, Intelligent Polymer Research Institute Wollongong Australia; ^7^ Discipline of Surgery School of Medicine, Western Sydney University Sydney Australia; ^8^ Microsoft Ltd Australia Auckland New Zealand; ^9^ Agency for Clinical Innovation, NSW Health Sydney Australia; ^10^ School of Health Sciences, University of Sydney Sydney Australia; ^11^ System Sustainability and Performance, NSW Government Sydney Australia; ^12^ Australian Government Department of Health and Aged Care Canberra Australia

**Keywords:** artificial intelligence, medical devices, privacy, regulation, safety

## Abstract

Artificial Intelligence (AI) is generally defined as the development of computer systems or machines that can perform tasks typically requiring human intelligence and is increasingly being used in modern healthcare. While, various AI systems have existed for decades, its scale in healthcare has been escalated by global crises such as the COVID‐19 pandemic and military conflicts, which has demanded rapid implementation of health system processes that improve efficiency in resource constrained environments. As AI‐enabled technologies gain prominence, it is vital for surgeons to understand the various types of AI systems and their applications in medical practice.

## 1 Introduction

Artificial Intelligence (AI) is generally defined as the development of computer systems or machines that can perform tasks typically requiring human intelligence and is increasingly being used in modern healthcare. While various AI systems have existed for decades, their scale in healthcare has been escalated by global crises such as the COVID‐19 pandemic and military conflicts, which have demanded rapid implementation of health system processes that improve efficiency in resource‐constrained environments. As AI‐enabled technologies gain prominence, it is vital for surgeons to understand the various types of AI systems and their applications in medical practice. AI systems are broadly categorized into different types, each focusing on different aspects of intelligence and problem‐solving [1, 2].

*Analytical AI* primarily addresses the challenge of understanding and processing data. This type of AI is particularly valuable in situations that require large volumes of data (e.g., images or text) to be analysed to extract meaningful insights. Some examples include Convolutional Neural Networks, Natural Language Processing, Data Mining, and Predictive Analytics. *Cognitive AI* is designed to enhance decision‐making, mirroring human cognitive processes such as perception, reasoning, and problem‐solving, and includes techniques such as Human‐Computer Interaction, Emotion AI, Speech Recognition, and Adaptive Learning Systems. *Generative AI* represents an advanced form of AI that focuses on problem‐solving and critical thinking. Unlike Analytical and Cognitive AI, Generative AI produces novel outputs such as text, images, and code. Notable examples include OpenAI's ChatGPT, which generates new text; DALL‐E, which creates new images; and GitHub Copilot, which assists in generating new code. These systems leverage complex models to generate content that was not explicitly programmed into them by their creators. *Humanoid AI*, poised to revolutionize clinical practice, is designed to interact with the environment and people, mimicking human abilities cognitively and in physical form. These AI systems combine advanced robotics with AI‐driven decision‐making, enabling them to perform tasks that require a high degree of precision, dexterity, and adaptability—traits traditionally associated only with humans. Lastly, *Agentic AI* refers to systems that can autonomously perform tasks and make decisions with minimal human intervention [3]. In the context of surgery, it has the potential to assist in complex procedures, enhancing precision, and reducing human error. By leveraging machine learning and real‐time data analysis, such systems could optimize surgical outcomes, predict complications, and provide personalized recommendations based on patient‐specific data. These could range from clinical workflow automation to multi‐agent aided diagnosis. While agentic AI holds promise for improved efficiency and safety, its implementation raises ethical concerns regarding autonomy, accountability, and the role of surgeons in decision‐making processes.

AI is currently integrated into healthcare to perform (a) service‐based roles, (b) in research and education, and (c) is incorporated into medical devices. The scale of AI applications in healthcare has challenged the regulatory and legal frameworks in which the health system functions, creating uncertainty for all those who engage with it. This includes device companies, software developers, healthcare professionals, educators, hospitals, and insurers. As governance and legislation in Australia adapt to this disruptive technology, the substantial benefits of AI are equally matched by the risks of misapplication, potentially eroding trust in the healthcare industry [4].

A risk assessment matrix is a commonly deployed tool in the health care industry to identify potential risks, categorise impact on patients, providers and organisations and develop appropriate implementation plans. Such tools are validated tools in health which comply with the International Organisation of Standards (ISO) 31 000 risk management framework [5]. Risk Matrices can list risks as quantitative (adding a numerical value to risk) or qualitative (high, medium and low) often coded in traffic light colours, which allows organisations to identify, analyse and evaluate risks and develop strategies to address them.

This paper is an interdisciplinary perspective on the evolving landscape of AI in healthcare using a traffic light framework of risk stratification to assist surgeons in making informed decisions so they can realize the benefits of AI without compromising the safety, confidentiality, and trust of their patients.

## 2 AI as a Service

Service‐based roles are often integrated into administrative tasks such as resource planning in hospitals, predicting and preventing adverse clinical events by incorporation into electronic medical records (EMRs) or generating consulting letters, thereby reducing the cognitive load on the workforce.

A rapidly increasing application of AI for Australian surgeons is in clinical transcription software. Here, the AI software serves as an ambient listening tool during doctor‐patient consultations to generate automated clinical letters. Legislation around obtaining consent from patients prior to recording the consultation by the AI scribes varies from state to state. With the exception of Northern Territory, Queensland, and Victoria, where consent is deemed prudent, in all other states and territories in Australia, consent is required for all parties prior to a recording even if the recording is deleted after the consultation [6].

In addition, it is important that surgeons understand that providers of such service‐based applications are not regulated under the Therapeutics Goods Act 1989 as these scribing applications do not meet the definition of a medical device unless they perform other functions [7]. In Australia, they are subject to the Privacy Act 1988 enforced by the Office of The Australian Information Commissioner [8]. These rules apply to all organisations that provide a health service or hold health information, irrespective of the size of the business. Practitioners and their organisations are liable if patient privacy is breached; therefore, surgeons need to be aware of data storage, transfer, processing, ownership, and downstream applications [9].

It may not be clear to clinicians or their patients where the data is being stored, who owns the data, how it will be used, and how privacy will be maintained. Many AI constructs are self‐learning, and data, particularly in open access web‐based platforms, may be returned to the vendor for training the algorithms. If undisclosed, this may breach privacy, exposing clinicians to breach of privacy laws and medicolegal liability. As a result, most jurisdictions recommend the use of vendors where there is no return of patient data to safeguard patient privacy and information. For surgeons who use such applications, it is important to demand transparency about data handling from software companies.

The accessibility and scale of AI accelerate the positive and negative impacts of software tools broadly. In 2021, the Australian Alliance for Artificial Intelligence in Healthcare (AAAiH) published a roadmap for AI in healthcare, identifying eight priority areas [10]. The highest priority areas for Australians were first, that AI is deployed safely and in an ethical manner, and second, it maintains the privacy and security of health information. Since service‐based AI products are most likely to be used in the outpatient clinic, are easier to access, and are not regulated in the rigorous way that medical devices are, a good understanding of the privacy principles is crucial for surgeons. Potentially, professional bodies such as colleges could support clinicians to acquire this knowledge through Continuous Professional Development (CPD) platforms.

## 3 AI in Medical Research

Whilst AI is the subject of much research in surgery, academic institutions have been challenged by the rapid rise in the use of generative AI as a methodological tool in research, questioning the definitions of original content, creativity, and problem‐solving capability among researchers.

In AI assisted literature reviews, there is a risk of bias, especially in areas where there is limited data. Artificial hallucination refers to potential misinformation that is relayed in an authoritative tone by generative AI models which may collate poor quality data to make scientific conclusions [11]. Therefore, the application of AI in research needs risk mitigation strategies. This bias may affect smaller subspecialties as well as research involving the evaluation of new technologies due to existing bias in the limited literature. AI can also potentiate social inequities by reducing the visibility of vulnerable populations, such as females, rural, culturally and linguistically diverse, indigenous, disabled and elderly individuals who may not be represented in the literature that the AI algorithms are trained to reference [12].

The attribution of AI‐generated contributions to intellectual property can also be challenging, which in research ranges from the writing of a manuscript to the inventive process of medical devices. A patentable invention is based on documented inventive steps. Therefore, whilst on one hand our educational bodies must develop governance strategies to authenticate authorship, additional change in intellectual property legislation is also required in medical device development and commercialisation, to prevent delay in medical innovations from being translated to the bedside.

## 4 AI in Medical Devices

In medical devices, AI can play a range of roles in diagnosis, treatment, or serve as virtual health assistants [13]. While AI can function as a medical device in its own right, it can also be integrated into other software‐ or hardware‐based medical devices, such as automated decision‐making devices (insulin pumps or fall monitors) or chatbots [14]. Whilst those products that are medical devices are regulated under the Therapeutic Goods Act 1989, the degree of regulation varies upon the risk or class of the device [15]. Surgeons are more likely to encounter higher risk devices (such as class III devices) in the operating theatre setting, rather than the outpatient clinic, and therefore, it is likely that these devices will have to undergo numerous levels of safety, efficacy, and cost‐effectiveness checks by federal and state governments, as well as hospitals and sometimes even individual departments before use. Low and medium risk devices (class I, IIa, and IIb), with less regulatory oversight, are more likely to be used by surgeons in the outpatient clinic, and therefore, education is important since, like any other tool, the user is ultimately responsible.

### 4.1 Regulation of AI in Medical Devices

Like all software‐based medical devices in Australia, according to the Therapeutics Goods Act 1989, Section 41BD, AI is regulated as a medical device if it is intended to be used for [16]:

- diagnosis, prevention, prediction, monitoring, prognosis of a disease, injury or disability
- alleviation of or compensation for an injury or a disability
- investigation of anatomical or physiological processes
- control or support of conception

This regulatory framework gives surgeons confidence that these products have been subjected to the necessary oversight.

*AI in Hardware‐Based Devices*: AI has the potential to influence each step of the process of hardware‐based medical devices from development to deployment. In silico medicine refers to the computer simulation of experiments that can accelerate research by predicting outcomes of research, thus reducing or replacing the traditional steps required for a medical device to get from bench to bedside. It can play a role in the discovery of new materials by developing optimal compositions for specific functions. It has the potential in the future to replace in vitro experiments, animal trials and, potentially in some instances, when a large enough sample size cannot be recruited, even clinical trials [17]. This has led governments in Europe and the United States to support international alliances to advise policymakers of being abreast of these developments in the pathway of medical device development and future regulation and deployment [18].

*AI in Software‐Based Products*: Whilst hardware is easier to regulate and its use easier to credential, software products add additional challenges since supply cannot be as readily controlled through jurisdiction boundaries. As with hardware medical devices, the risk of causing adverse outcomes to the patient will vary according to the intended purpose of the device and whether it is being used consistently with that intended purpose. For instance, a phone‐based 3D scanner used to generate prosthetics for a patient may have less adverse effect if the data used to generate the prosthetic is not complete or has poor resolution. Ultimately, the prosthetist can change the manufactured prosthetic and even so, it may not alter the patient's clinical outcome. However, if there is an error using AI optimised 3D scanning software in virtual planning and 3D printing an orthodontic device, inaccuracies may have more serious consequences for the patient.

Breast screening is an example where a software‐based AI application has been studied in depth to perform numerous functions. In screening mammography, it has shown a high false positive rate compared to humans [19]. If AI is used as an assistive device for the radiologist, then there arises a risk of automation bias—humans may assume that the machine is correct or fear medicolegal or ethical risks if they ignore the machine's warning [19]. This can ultimately increase the workload of an already overburdened workforce and lead to unnecessary use of healthcare resources, such as additional pathology or radiology tests. These additional tests may be associated with additional risks to the patient. However, in breast screening, AI has demonstrated benefit in replacing the role of a second radiologist for double reading in breast screening [20, 21] which is valuable in areas with a limited workforce. Despite this benefit, patients have reported that they must be informed regarding the use of AI applications in their healthcare to ensure acceptability and maintain trust [22].

When applying these tools, clinicians must understand whether the sample used to train the AI represents the patient population to which it is applied. For instance, an AI model trained on a sample of mammograms of a particular country where breast cancer prevalence is low may not yield the same accuracy in another country where the prevalence is high. For a time‐constrained workforce, this may not be transparent or easy to comprehend. Therefore, clinicians must treat AI‐based devices like any other medical device and ensure their use is consistent with the intended purpose it was approved for.

Chatbot technology is another example of a software‐based AI device. The rise of deep fake technology, which can create realistic avatars of a clinician's face and voice, poses challenges in the era of telehealth acceptance. Healthcare consumers must be able to discern whether they are interacting with a real human or a deep fake chatbot that resembles their doctor. This distinction is crucial for understanding who is collecting their medical data and dispensing medical advice. In Australia, chatbots may be regulated as medical devices, but ensuring acceptability and transparency among healthcare consumers is essential. Without these assurances, trust in healthcare can be eroded despite regulatory measures. However, whilst telehealth is a medium to dispense a health service, AI chatbots are medical devices that perform a service. A potential risk is that they can be accessed by Australian patients over the internet even if they have not obtained regulatory approval in Australia. Australian patients who access telehealth services from overseas practitioners generally cannot obtain medications or procedures unless the patient physically travels overseas to the jurisdiction where the doctor is accredited to practice. This is because, in most jurisdictions, there is a separation between the person prescribing and the person dispensing the drug. However, companies providing the AI platforms can integrate the functions of the prescriber, dispenser, and the courier, which exposes patients to different risks warranting a review of the potential conflicts of interest by integrating these services more readily [23]. Patients who have reduced access to healthcare services, such as rural patients or patients with poor health literacy, are more vulnerable to such misuse. Therefore, consumers, clinicians, hospitals, manufacturers, and government all have a role to help mitigate harm. The integration of AI into medical practice necessitates robust governance frameworks, which require collaboration of numerous federal and state agencies as well as hospitals and individual clinical practices that are responsible for procurement and implementation of these products, to maintain trust and ensure that AI applications are used ethically and transparently. As these products evolve and get integrated into clinical practice, the potential role of the Australian Health Practitioner Regulation Agency to collaborate with national peak educational bodies, such as colleges, to monitor changes to health service delivery and credentialing practitioners in the use of such software also becomes more important [14].

## 5 A Proposed Strategy for Clinical Practice

Surgeons need to enhance their awareness of the extent to which AI is integrated into their clinical practice and understand the relevant Australian governance framework that applies in Australia. To address this knowledge gap, we propose a ‘traffic light system’ to categorise AI applications according to the types of risk they pose and potential indicators of creep between the categories.

### 5.1 Green: Low‐Risk Applications

Low‐risk applications are mainly applicable to the business or administrative side of healthcare and applications that are not directly involved in providing some form of therapy or influencing clinical decision making. This includes services‐based examples discussed above, where the risks are more likely to be administrative without a high impact on clinical outcome. However, there is still a risk that errors can still have a negative impact based on the scale of their application. For instance, if an AI software was deployed to assist in procurement at a hospital, an error may lead to the delivery of health care to patients at the institution. However, if the same error was made by a state‐based procurement system, that impact would be larger. In addition, as software evolves, functionality creep into the iteration of existing systems can blur the lines between low and medium risk platforms.

### 5.2 Amber: Medium‐Risk Applications

This category includes areas of applications that are starting to integrate into clinical care but have very low risk of harm, for instance if AI is used to prolong battery life within a device or the introduction of AI to replace the need for a second reader for screening mammography where benefit has been established.

Use of AI in medical research may fall under this category depending on the intention and impact of this research. For instance, a publication evaluating the feasibility of AI in a particular health intervention is of low risk; however, once the impact of that research is translated into a change in practice or change in policy, it is important to minimise bias to negatively influence health decisions.

Clinical decision support software also falls under this category, including those applications that are exempt from regulation, simply because the risk to the patient may be low, but because of less regulatory oversight, clinicians need to be more educated and proactive in managing the risks to themselves. To be exempt from regulation in Australia, clinical decision support software must meet all three criteria listed below [24]:

1. does NOT directly process or analyse a medical image or a signal from another medical device (including an in vitro diagnostic device); and
2. is solely used to provide or support a recommendation to a health professional about prevention, diagnosis, curing, or alleviating a disease, ailment, defect, or injury; and
3. does NOT replace the clinical judgment of a health professional in relation to making a clinical diagnosis or decision about the treatment of patients.

### 5.3 Red: High‐Risk Applications

High risk applications are applications that play a more direct role in clinical care. Typically, they carry more regulatory burden and are more likely to come under a high degree of regulatory governance, though as evidence, regulation, governance, and thereby safety matures, their risk classification may be revised in the future. These include examples such as chatbots or hardware with AI integrated into them. Clinicians are more likely to encounter high risk applications in the operating theatre and therefore, the burden of governance is more likely to be shared by the individual surgeon as well as the hospital. In Australia, any AI‐based application that otherwise fulfills the role of a medical device and has not received regulatory approval for its intended use cannot be used in clinical care unless it is exempt from regulation. Approval in another jurisdiction, even with good evidence of benefit, does not grant clinicians the authority to use it in Australia.

There are two exceptions to this rule, similar to any other Therapeutic good:

1. The clinician must seek special access scheme or authorized prescriber approval from the TGA.
2. Enrol in a clinical trial governed by a human research ethics committee, and notification to the TGA (clinical trials notification) is provided by the sponsor prior to trial commencement.

However, even within the context of a clinical trial, ethics committees must also be supported by experts to clarify who is collecting the data and review the future use of data, which is often commercial property of AI companies. This is particularly important for Australia, which has seen considerable success in attracting clinical trials in recent years. Therefore, ethics committees need extended expertise to develop robust data governance when reviewing applications for AI‐based trials. The National Health and Medical Research Council (NHMRC) is best placed to provide this guidance to ethics committees about acceptable standards for AI‐based human research [14].

By implementing this traffic light coding system, we can provide surgeons with guidance to navigate the complexities of AI integration in healthcare, promoting safety, transparency, and trust in these evolving technologies.

## 6 Cost and Value

Responsible integration of AI needs good governance. However, data governance is costly, and in Australia's fragmented health system, governance measures are replicated at multiple levels between states and across institutions with additive costs but without added benefit. Achieving cost‐effectiveness and thorough regulatory analysis simultaneously is challenging without national and system‐wide collaboration, necessitating a strategic approach that encompasses privacy, intellectual property, credentialing, education of practitioners, changes to health services delivery, and regulation of devices. Additionally, establishing clear policies for data governance and security is crucial to maintain trust and integrity in AI applications.

Cost of maintaining and providing AI services will also impact Australian innovation, increasing overseas dependency, which may influence the Australian Medical Technology ecosystem and have downstream impacts on whether Australia becomes a consumer or creator of AI‐based systems? By fostering a coordinated, collaborative, and forward‐thinking approach, Australia can maximize the benefits of AI in healthcare while safeguarding its national interests and patient data sovereignty. This will also improve access to new emerging AI systems, improving Australia's position at the global research table and improving the ability to generate protectable intellectual property.

## 7 Conclusion

The integration of AI in healthcare presents both remarkable opportunities and significant challenges. For AI to be effectively and safely integrated at a health system level, a comprehensive strategy involving rigorous regulatory oversight, robust data governance, and strategic investment is essential. Collaboration at all levels of the health sector—micro (clinician leadership), meso (hospital and local health districts) and macro (state and federal bodies) are necessary for effective implementation. By fostering a coordinated, transparent, and ethically grounded approach, the healthcare industry can ensure that AI serves to enhance patient care, improve health outcomes, and maintain public trust.

Surgeons are at the coalface of patient care and can play a leadership role in the responsible implementation of AI in their teams, maintaining patient trust. They must be adequately informed about the extent of AI integration and its implications for clinical practice. The proposed traffic light coding system offers a practical framework to start navigating AI applications, assisting surgeons in making informed, risk‐based decisions. The coding system emphasises that service‐based applications of AI are more likely to be encountered in the outpatient clinic. They are lower risk (green) but conversely, require a higher degree of scrutiny by clinicians to minimise risk to the privacy of patient information and also ensure it will not bias their decision making through scope creep. Higher risk applications (red) are subject to more rigorous oversight with numerous levels of governance. Surgeons are more likely to use it in the operating room environment. However, these products can also be accessed in the outpatient clinic and products that have less risk to patients in our current framework also have less stringent regulatory burdens or may be exempted from regulation. It is important for surgeons to understand the existing regulatory frameworks in Australia governing AI, so they can manage their own personal risk as well as clinical risks as they implement and apply such technologies in situations where there is less system‐level oversight. In between, the amber, is a sliding scale where risk depends on the intention of use of the tool. Surgeons need to be aware that products that have a lower level of regulation require a higher need for individual understanding, as the risk and responsibility of the product is ultimately in the hands of the user.

## Author Contributions

**Payal Mukherjee:** conceptualization, writing – original draft. **Amin Beheshti:** writing – original draft, conceptualization. **Shivani Angelique Kumar:** data curation, investigation, writing – review and editing. **Gordon Wallace:** conceptualization, writing – review and editing. **Neil Merrett:** writing – review and editing. **Jonathan Clark:** writing – review and editing, conceptualization. **Simon Kos:** writing – review and editing. **Ellen Rawstron:** writing – review and editing. **Jian Yang:** writing – review and editing. **Stuart Grieve:** writing – review and editing. **Amith Shetty:** writing – review and editing, supervision, writing – original draft. **Simon Singer:** writing – review and editing, supervision, conceptualization.

## References

[1] A. Beheshti , “Empowering Generative AI With Knowledge Base 4.0: Towards Linking Analytical, Cognitive, and Generative Intelligence. IEEE International Conference on Web Services IEEE ICWS 2023”.

[2] A. Beheshti , J. Yang , Q. Z. Sheng , et al., “ProcessGPT: Transforming Business Process Management With Generative Artificial Intelligence. 2023 IEEE International Conference on Web Services IEEE ICWS 2023,”.

[3] J. Qiu , K. Lam , G. Li , et al., “LLM‐Based Agentic Systems in Medicine and Healthcare,” Nature Machine Intelligence 6 (2024): 1418–1420.

[4] Unlocking the Power of AI to Transform Healthcare (Department of Health and Aged Care, 2024).

[5] G. Pascarella , M. Rossi , E. Montella , et al., “Risk Analysis in Healthcare Organizations: Methodological Framework and Critical Variables,” Risk Management and Healthcare Policy 14 (2021): 2897–2911, 10.2147/RMHP.S309098. 34267567 PMC8275831

[6] Avant Group Limited , Artificial Intelligence for Medical Documentation (Avant Mutual Group Limited, 2024), https://avant.org.au/resources/artificial‐intelligence‐for‐medical‐documentation.

[7] Goods Act , “Therapeutic Goods Act 1989,” 2024.

[8] Office of the Australian Information Commissioner , Health and Medical Research (Australian Government, 2024), https://www.oaic.gov.au/privacy/privacy‐legislation/the‐privacy‐act/health‐and‐and‐medical‐research.

[9] S. Qin , B. Chislett , J. Ischia , et al., “ChatGPT and Generative AI in Urology and Surgery—A Narrative Review,” BJUI Compass 5, no. 9 (2024): 927–935. 10.1002/bco2.390 PMC11420103 39323919

[10] Australian Alliance for Artificial Intelligence in Healthcare , “A Roadmap for Artificial Intelligence in Healthcare for Australia Sydney,” 2021.

[11] H. Alkaissi and S. I. McFarlane , “Artificial Hallucinations in ChatGPT: Implications in Scientific Writing,” Cureus 15, no. 2 (2023): e35179, 10.7759/cureus.35179. 36811129 PMC9939079

[12] M. Mittermaier , M. M. Raza , and J. C. Kvedar , “Bias in AI‐Based Models for Medical Applications: Challenges and Mitigation Strategies,” Npj Digital Medicine 6, no. 1 (2023): 113. 37311802 10.1038/s41746-023-00858-z PMC10264403

[13] J. J. Wadden , “Defining the Undefinable: The Black Box Problem in Healthcare Artificial Intelligence,” Journal of Medical Ethics 48, no. 10 (2022): 764–768. 10.1136/medethics-2021-107529 34290113

[14] Research Australia , “Positioning Australia as a Leader in Digital Economy Regulation (Automated Decision Making and AI Regulation): Issues Paper. Canberra,” 2022.

[15] Therapeutic Goods Administration , What Classification Is My Medical Device? (Department of Health and Aged Care, 2024), https://www.tga.gov.au/resources/what‐classification‐my‐medical‐device.

[16] Therapeutic Goods Administration , Regulation of Software Based Medical Devices Australia (Department of Health and Aged Care, 2024).

[17] O. Faris and J. Shuren , “An FDA Viewpoint on Unique Considerations for Medical‐Device Clinical Trials,” New England Journal of Medicine 376, no. 14 (2017): 1350–1357. 28379790 10.1056/NEJMra1512592

[18] Avicenna Alliance , “Our Mission Belgium 2024,” https://www.avicenna‐alliance.com/our‐mission.html.

[19] S. Bhoi , A. Singh , T. Sinha , et al., “Magnitude and Spectrum of Injuries Sustained in Road Traffic Accidents Among Two Wheeler Riders and Correlation With Helmet Use,” Journal of Emergencies, Trauma, and Shock 11, no. 3 (2018): 160–164. 30429621 10.4103/JETS.JETS_119_17 PMC6182975

[20] A. Y. Ng , C. J. G. Oberije , É. Ambrózay , et al., “Prospective Implementation of AI‐Assisted Screen Reading to Improve Early Detection of Breast Cancer,” Nature Medicine 29, no. 12 (2023): 3044–3049. 10.1038/s41591-023-02625-9 PMC10719086 37973948

[21] K. Lång , V. Josefsson , A.‐M. Larsson , et al., “Artificial Intelligence‐Supported Screen Reading Versus Standard Double Reading in the Mammography Screening With Artificial Intelligence Trial (MASAI): A Clinical Safety Analysis of a Randomised, Controlled, Non‐Inferiority, Single‐Blinded, Screening Accuracy Study,” Lancet Oncology 24, no. 8 (2023): 936–944. 37541274 10.1016/S1470-2045(23)00298-X

[22] S. M. Carter , L. Carolan , Y. Saint James Aquino , et al., “Australian Women's Judgements About Using Artificial Intelligence to Read Mammograms in Breast Cancer Screening,” Digital Health 9 (2023): 20552076231191057. 37559826 10.1177/20552076231191057 PMC10408316

[23] M. Clark and S. Bailey , “Canadian Agency for Drugs and Technologies in Health,” in Chatbots in Health Care: Connecting Patients to Information: Emerging Health Technologies, vol. 4 (Canada's Drug Agency, 2024), https://www.ncbi.nlm.nih.gov/books/NBK602381/. 38564540

[24] Therapeutic Goods Administration , Clinical Decision Support Software (Department of Health, 2021).

